# Association of antidepressants with brain morphology in early stages of psychosis: an imaging genomics approach

**DOI:** 10.1038/s41598-019-44903-y

**Published:** 2019-06-11

**Authors:** Oleg Bykowsky, Fabienne Harrisberger, André Schmidt, Renata Smieskova, Daniel J. Hauke, Laura Egloff, Anita Riecher-Rössler, Paolo Fusar-Poli, Christian G. Huber, Undine E. Lang, Christina Andreou, Stefan Borgwardt

**Affiliations:** 1 Neuropsychiatry and Brain Imaging, Department of Psychiatry (UPK), University of Basel, Basel, Switzerland; 2 Psychiatric University Hospital (UPK), University of Basel, Basel, Switzerland; 3 Early Psychosis: Interventions and Clinical Detection (EPIC) lab, Department of Psychosis Studies, Institute of Psychiatry, Psychology & Neuroscience, King’s College London, London, United Kingdom; 4 Department of Mathematics and Computer Science, University of Basel, Basel, Switzerland; 5 Center for Addiction Medicine, Châlons-en-Champagne General Hospital, Châlons-en-Champagne, France

**Keywords:** Cognitive neuroscience, Neurology

## Abstract

Depressive symptoms in subjects at Clinical High Risk for Psychosis (CHR-P) or at first-episode psychosis (FEP) are often treated with antidepressants. Our cross-sectional study investigated whether brain morphology is altered by antidepressant medication. High-resolution T_1_-weighted structural MRI scans of 33 CHR-P and FEP subjects treated with antidepressants, 102 CHR-P and FEP individuals without antidepressant treatment and 55 controls, were automatically segmented using Freesurfer 6.0. Linear mixed-effects modelling was applied to assess the differences in subcortical volume, surface area and cortical thickness in treated, non-treated and healthy subjects, taking into account converted dosages of antidepressants. Increasing antidepressant dose was associated with larger volume of the pallidum and the putamen, and larger surface of the left inferior temporal gyrus. In a pilot subsample of separately studied subjects of known genomic risk loci, we found that in the right postcentral gyrus, the left paracentral lobule and the precentral gyrus antidepressant dose-associated surface increase depended on polygenic schizophrenia-related-risk score. As the reported regions are linked to the symptoms of psychosis, our findings reflect the possible beneficial effects of antidepressant treatment on an emerging psychosis.

## Introduction

Appearance of psychosis is considered to be the driving force of multiple debilitating mental disorders, including schizophrenia^1,2^, which affects 0.7% of the world population^3^. Psychosis presents itself in a diffuse temporal and phenomenological range of symptoms, of disparate severity and persistence^4,5^. Before their assignation to the first episode of psychosis (FEP) group, subjects at risk belong to a heterogeneous group of Clinical High Risk for Psychosis (CHR-P), characterised by attenuated psychotic symptoms, brief limited intermittent psychotic symptoms, genetic risk or schizotypal traits and a decline in social and occupational functioning^6–9^. The disorder usually manifests itself in early adulthood and the active search for reliable transition markers continues as around 20% of the CHR-P undergo transition to FEP during the first two years^10^.

Multiple findings indicate that CHR-P and FEP show similar functional^11^ and structural brain abnormalities^9,10,12,13^, but it is unclear to what extent they reflect genetics, general distress, medication effects or are unique features associated with the at risk stage of the disorder. The psychosis vulnerability seems associated with progressive nonlinear morphological changes, to which the pleiotropic genetic factors, the medication and non-genetic factors may contribute^14–16^. CHR-P subjects show structural abnormalities in frontal, cingular and temporal cortices, insular regions, temporal gyrus and some of these changes may be predictive of later transition to psychosis^16–24^.

CHR-P and FEP individuals often suffer from comorbid depressive and anxiety symptoms^25,26^ that can precede^27^ or accompany the onset of attenuated positive psychotic symptoms^9,28^. These features are the main reason for seeking help at specialized services^29^ and the occurrence of these is significantly associated with decreased likelihood of remission from CHR-P^30,31^. Antipsychotic treatment usually begins after the first episode of psychosis and non-random comparative studies of antidepressants and antipsychotics indicate that antidepressants might be a beneficial effective treatment for preventing psychosis in CHR-P individuals^32–34^.

Currently CHR-P and FEP subjects are occasionally treated with antidepressants if they present depressive symptoms^35^, but the effect of antidepressant medication on brain morphology has, to the best of our knowledge, rarely been explored in the CHR-P and FEP cohorts.

The morphological reduction in major depression was observed in the prefrontal, temporal and parieto-occipital regions, basal ganglia, inferior/middle temporal gyri, hippocampus, and the cerebellum^36–39^. It was shown for depression that antidepressant treatment was connected to enlargement of the frontal cortex, middle frontal gyrus, anterior cingulate, and hippocampus^40–44^. It is linked to an increase in grey matter volume^45^, it influences brain connectivity^46^, and it modulates synaptogenesis, neurogenesis and dendritic arborisation in animal models and in the human hippocampus^47–50^. A recent study of affective psychoses^51^ did not detect long-term antidepressant effect on any brain regions, although – like in other studies – only general presence or absence of antidepressants was used as a predictor.

As the goal of ongoing international research efforts is to establish reliable brain markers to complement clinical features for the prediction of psychosis onset, it is crucial to disentangle disease and medication effects on brain morphology in CHR-P and FEP individuals. Therefore, the aim of our investigation was to study whether morphological features such as cortical thickness, surface area and subcortical volume were altered after administration of antidepressant medication in CHR-P and FEP subjects. The focus on subcortical volumes is based on previous studies in FEP^14,52,53^ and UHR^54,55^ patient showing alterations in these cohorts relative to healthy volunteers. Cortical thickness and surface area were further considered because such surface-based measures are probably more sensitive to detect brain alterations in early phases of psychosis^56^.

As there are only a few studies showing an impact of antidepressant medication on volume, thickness and surface^26,51^ in CHR-P and FEP, we investigated the influence of antidepressant dose on the whole-brain. As shown in the cited literature, antidepressant effect is often linked to modifications in the frontal regions and in hippocampus, and we expected to register the structural effects primarily in these regions.

While in CHR-P and FEP the transition risk and morphology^57^ are co-modulated by genetics, our additional aim was to identify if there is an impact of combined cumulative estimate of known genomic risk loci (*polygenic schizophrenia-related risk scores* or *PSRS*^58^) on the effect of antidepressants on brain morphology in CHR-P and FEP, as subjects with high/low genetic risk might have different medication effect because of the different illness endophenotypes or a genetic overlap between pathogenesis and drug action^59^. Although a recent study^60^ did not find evidence of genetic overlap between schizophrenia risk and subcortical volume, several studies have already reported an association of a PSRS with brain volume^61–63^. Our focus on analysing the interaction of PSRS and antidepressant medication on thickness, surface and subcortical volume is novel in the context of previous studies and we hypothesise that the antidepressant medication effect on brain morphology may depend on the genetic predisposition for psychosis.

## Results

### Demographics and clinical characteristics

Samples with and without genetics data (Table 1) exhibited no significant differences *(p* > *0*.*05*) in age, *handedness or IQ*. SANS did not differ between CHR-P and FEP. There were significant differences between groups in *sex* distribution in the full sample (*p* < *0*.*001)*, but not in the PSRS subsample (*p* > *0*.*05)*, and in the *years of education* distribution in the full sample *(p* < *0*.*001)*, but not in the PSRS subsample *(p* > *0*.*05)*. In both samples, there was a significant between-group difference in *overall BPRS (both samples p* < *0*.*001)*, *cannabis consumption* (*p* = *0*.*007*, *p* < *0*.*001*) and *GAF (both samples p* < *0*.*001)*.

**Table 1:** Demographics and clinical characteristics.

	Combined CHR-P and FEP without antidepressants (NT)	Combined CHR-P and FEP treated with antidepressants (T)	HC	Statistics	P-value	Post-hoc
**Number of subjects**						
PSRS subsample	43	15	14			
Full sample	102	33	55			
**Sex M/F**						
PSRS subsample	‘23/10	‘12/3	‘6/8	X^2^ = 4.43 (2)	0.11	
Full sample	‘71/31	‘23/10	‘22/33	X^2^ = 14.2 (2)	0.0008	
**Mean age in years (s**.**d**.**)**						
PSRS subsample	24.58 (6)	26.87 (7.23)	26.4 (2.75)	Anova F value= 1 (2)	0.37	
Full sample	25.29 (6.13)	26.55 (6.56)	25.93 (4.68)	Anova F value= 0.64 (2)	0.53	
**Handedness left/non-left**						
PSRS subsample	‘6/37	‘2/13	‘1/13	X^2^ = 0.516 (2)	0.77	
Full sample	‘10/91	‘2/31	‘3/35	X^2^ = 0.306 (2)	0.86	
**Years of education (s**.**d**.**)**						
PSRS subsample	13.58 (2.6)	14.33 (3.36)	16.1 (3.4)	Anova F value= 2.08 (2)	0.13	
Full sample	13.23 (2.6)	14.24 (3.23)	15.32 (2.76)	Anova F value = 8.22 (2)	0.0004	HC > NT
**IQ (s**.**d**.**)**						
PSRS subsample	108.70 (14.53)	115.33 (19.18)	117.6 (11.2)	Anova F value = 1.99 (2)	0.15	
Full sample	108.44 (15.02)	113.79 (16.15)	116.5 (11.43)	Anova F value = 2.87 (2)	0.061	
**BPRS (s**.**d**.**)**						
PSRS subsample	45.46 (15.17)	41 (10.91)	24.4 (0.91)	Anova F value = 14.4 (2)	<0.0001	HC < T, NT
Full sample	42.63 (13.57)	40.67 (9.09)	24.16 (0.57)	Anova F value = 56.1 (2)	<0.0001	HC < T, NT
**BPRS affective (s**.**d**.**)**						
PSRS subsample	6.54 (3.44)	7.07 (2.53)	3.20 (0.56)	Anova F value = 8.44 (2)	0.0006	HC < T, NT
Full sample	6.02 (2.90)	7.03 (2.61)	3.09 (0.35)	Anova F value = 36.6 (2)	<0.0001	HC < T, NT
**BPRS negative (s**.**d**.**)**						
PSRS subsample	6.15 (3.07)	6.21 (2.64)	3 (0)	Anova F value = 7.93 (2)	0.0008	HC < T, NT
Full sample	5.25 (2.81)	6.23 (2.66)	3 (0)	Anova F value = 24.3 (2)	<0.0001	HC < T, NT
**BPRS positive (s**.**d**.**)**						
PSRS subsample	8.56 (4.52)	7.21 (4.37)	3 (0)	Anova F value = 9.95 (2)	0.0002	HC < T, NT
Full sample	8.07 (4.52)	6.80 (3.63)	3 (0)	Anova F value = 34.7 (2)	<0.0001	HC < T, NT
**BPRS activation (s**.**d**.**)**						
PSRS subsample	3.85 (1.87)	3 (0.78)	3 (0)	Anova F value = 2.65 (2)	0.079	
Full sample	3.85 (1.92)	3.23 (0.97)	3 (0)	Anova F value = 6.43 (2)	0.002	HC < NT
**SANS (s**.**d**.**)**						
PSRS subsample	17.61 (14.38)	18.71 (12.53)	NA	t = 1.4	0.2	
Full sample	14.10 (12.64)	17.70 (12.42)		t = 0.27	0.8	
**GAF (s**.**d**.**)**						
PSRS subsample	64.81 (17.32)	63.93 (13.14)	89.3 (4.9)	Anova F value = 15.8 (2)	<0.0001	HC > T, NT
Full sample	65.11 (15.96)	60.81 (13.16)	91.9 (4.5)	Anova F value = 89 (2)	<0.0001	HC > T, NT
**Antipsychotics no/yes**						
PSRS subsample	35/8	11/4	NA	X^2^ = 0.086 (1)	0.08	
Full sample	73/29	24/9		X^2^ ~ 0 (1)	~1	
**Cannabis no/yes/NA**						
PSRS subsample	34/9/0	6/8/1	13/1/0	X^2^ = 9.95 (2)	0.0069	
Full sample	80/21/1	17/13/3	52/2/1	X^2^ = 20.9 (2)	<0.0001	
**Normalized PSRS (s**.**d**.**)**						
PSRS subsample	0.02 (1)	0.02 (1.05)		Anova F value = 0.09 (2)	0.92	

sd: standard deviation; T: treated by antidepressants; NT: not treated by antidepressants; NA: no data; all post-hoc analyses correspond to p < 0.05.

### Morphometric differences

We proceed reporting effects that survived FDR correction with a significance threshold of p < 0.05 followed by exploratory findings that survived an uncorrected threshold of p < 0.01. Table 2 shows a summary of the significant LME results for antidepressants dose effects on brain regions in the whole sample; Table 3 shows the regions for which an interaction between PSRS and medication was required to explain variations in surface or volume.

**Table 2:** Summarized linear mixed effects models for antidepressants.

	Fusiform gyr. surf.		GP vol.		Inferior parietal lob. surf.		Inferior temporal gyr. surf.		MTG surf.		NAcc vol.		Putamen vol.	
	F	P-value	F	P-value	F	P-value	F	P-value	F	P-value	F	P-value	F	P-value
**FEP**, **CHR-P**, **HC**														
Age	0.0051	0.9434	0.0053	0.942	0.8376	0.3613	2.3312	0.1285	7.1333	0.0082***	2.733	0.1	0.724	0.3959
Antidepressants dose	7.5094	0.0067*	10.2445	0.0016***	8.0283	0.0051*	10.4924	0.0014***	7.8861	0.0055*	8.8841	0.0033*	9.9351	0.0019***
Antidepressants dose x hemisphere	0.9359	0.3346	0.1058	0.7454	1.9489	0.1644	1.0673	0.3029	0.0466	0.8294	0.2529	0.6156	0.5055	0.478
Diagnosis	3.7807	0.0246	2.2706	0.1061	0.0022	0.9978	5.1127	0.0069*	0.1802	0.8352	2.2488	0.1084	2.4659	0.0877
Hemisphere	0.017	0.8965	0.4368	0.5095	0.0481	0.8266	0.0013	0.9716	0.67	0.4142	0.0015	0.9697	0.0908	0.7635
Sex	5.1986	0.0237	9.8685	0.002***	1.1203	0.2912	4.0807	0.0448	1.1284	0.2895	1.0903	0.2978	6.8627	0.0095***
**FEP**, **CHR-P**														
Age	1.2241	0.2706	0.4671	0.4956	0.0062	0.9376	0.085	0.7711	4.1447	0.0438	1.6135	0.2063	0.1921	0.6619
Antidepressants dose	7.0187	0.0091*	10.8786	0.0013***	7.2448	0.008*	9.3836	0.0027*	7.2142	0.0082*	8.0509	0.0053*	11.2135	0.0011***
Antidepressants dose x hemisphere	0.9548	0.3303	0.3089	0.5793	2.1568	0.1443	1.378	0.2426	0.1566	0.693	0.0884	0.7667	0.4115	0.5223
Diagnosis	3.2054	0.0757	4.2515	0.0412	0.0094	0.9228	9.2156	0.0029*	0.1988	0.6564	1.3446	0.2484	2.4243	0.1219
Hemisphere	0.0104	0.9188	0.0043	0.9478	0.1529	0.6964	0.0797	0.7781	1.0156	0.3156	0.1842	0.6685	0.0027	0.9588
Sex	4.3878	0.0381	13.6249	0.0003***	3.3	0.0716	3.1462	0.0785	0.4173	0.5195	1.3707	0.2438	12.7727	0.0005***

*P-value < 0.01, ***survived correction for FDR with P-value < 0.05, GP: Globus Pallidus, MTG: medial temporal gyrus, NAcc: nucleus accumbens, gyr.: gyrus, lob.: lobule, surf: surface, vol.: volume.

**Table 3:** Summarized linear mixed effects models for antidepressants x PSRS interaction in the PSRS subsample.

	Lat. OFC surf.		Paracentral lob. surf.		Postcentral gyr. surf.		Precentral gyr. surf.	
	F	P-value	F	P-value	F	P-value	F	P-value
**FEP**, **CHR-P**, **HC with PSRS**								
Age	4.6873	0.0341	0.869	0.3548	3.3973	0.07	0.517	0.4748
ADeq x PSRS	7.0471	0.0095*	10.8924	0.0014*	9.3919	0.0028*	9.848	0.0023*
ADeq x PSRS x hemisphere	4.4517	0.0384	2.5105	0.1177	0.2056	0.6517	0.0081	0.9286
Antidepressants dose	2.9211	0.0923	3.5752	0.0632	4.08	0.0477	4.7202	0.0335
Diagnosis	0.0452	0.9559	0.5532	0.5778	0.2971	0.744	0.5067	0.6049
Hemisphere	0.1085	0.7428	0.2191	0.6412	0.0045	0.9466	0.1297	0.7198
PSRS	0.0187	0.8917	0.1503	0.6996	0.5883	0.4459	0.474	0.4937
Sex	1.1951	0.2784	0.0795	0.7788	0.0593	0.8085	0	0.9999
**FEP and CHR-P with PSRS**								
Age	3.8094	0.0565	0.6519	0.4232	4.1523	0.0468	0.4273	0.5163
ADeq x PSRS	7.0548	0.0098*	10.3231	0.002***	11.4315	0.0011***	10.5822	0.0017***
ADeq x PSRS x hemisphere	4.2635	0.0436	2.4821	0.121	0.1726	0.6795	0.0039	0.9504
Antidepressants dose	2.8239	0.099	3.0493	0.0869	4.1067	0.048	4.1415	0.0471
Diagnosis	0.0009	0.9765	0.6933	0.4089	0.0878	0.7681	0.5546	0.4599
Hemisphere	0.0433	0.8359	0.1112	0.7401	0.0638	0.8015	0.0194	0.8896
PSRS	0.1816	0.6718	0.2093	0.6493	1.8401	0.1808	1.5295	0.2219
Sex	0.6018	0.4415	0.0055	0.9412	1.0283	0.3154	0.1753	0.6772

*P-value < 0.01, ***survived correction for FDR with P-value < 0.05, OFC: orbito-frontal cortex, lat.: lateral, surf: surface, vol.: volume, ADeq: antidepressant medication fluoxetine equivalents, PSRS: polygenic schizophrenia risk score.

### Subcortical volume

We detected a significant main effect of antidepressant dosage on the *putamen (β* = *0*.*0166*, *s*.*e*. = *0*.*0053*, *p* = *0*.*0019*) and *the pallidum (β* = *0*.*0157*, *s*.*e*. = *0*.*0049*, *p* = *0*.*0016*), both survived FDR correction. A Tukey post-hoc test revealed an enlargement trend in antidepressant-treated CHR-P and FEP subjects’ subcortical volume. The *left pallidum* in the group of antidepressant-treated CHR-P and FEP subjects was enlarged compared to healthy controls (*p* = *0*.*04*). The *putamen* was enlarged in antidepressant-treated CHR-P and FEP subjects compared to the non-treated CHR-P and FEP subjects (left *p* = *0*.*05*, right *p* > *0*.*05*) and healthy controls (left *p* = *0*.*003*, right *p* = *0*.*006*).

A group-wise Tukey post-hoc test showed that the medicated CHR-P group exhibited significant enlargement in the *left putamen* (*p* = *0*.*02*) compared to unmedicated CHR-P and the medicated FEP group exhibited enlargement of the *right putamen (p* = *0*.*02*) and *bilateral pallidum (left p* = *0*.*006*, *right p* = *0*.*036*) compared to the unmedicated FEP.

We also found the main effect of antidepressant dosage on nucleus *accumbens* (*NAcc*) *(β* = *0*.*0152*, *s*.*e*. = *0*.*0051*, *p* = *0*.*0033)*, but it did not survive the FDR correction. A Tukey post-hoc test revealed that *left NAcc* was enlarged in the group of antidepressant-treated CHR-P and FEP subjects compared to the non-treated CHR-P and FEP subjects (*p* = *0*.*02*). There was a trend to enlargement in *left NAcc* in medicated FEP group (*p* = *0*.*03*) - compared to the unmedicated group.

We found no significant interaction effects of antidepressant dosage and PSRS for subcortical structure volume.

### Surface area

We detected a significant main effect of antidepressant dosage on the surface of the *inferior temporal gyrus* (*β* = *0*.*0154*, *s*.*e*. = *0*.*0048*, *p* = *0*.*0014*, *survived FDR*). For the surface of the *left inferior temporal gyrus* the Tukey post-hoc test revealed the significant enlargement (*p* = *0*.*01*) in antidepressant-treated CHR-P and FEP subjects compared to non-treated CHR-P and FEP subjects.

We detected a significant main effect of antidepressant dosage on the surfaces of the *fusiform gyrus (β* = *0*.*0134*, *s*.*e*. = *0*.*0049*, *p* = *0*.*0067)*, the *middle temporal gyrus* (*MTG*) (*β* = *0*.*0151*, *s*.*e*. = *0*.*0054*, *p* = *0*.*0055*), and the *inferior parietal lobule* (*β* = *0*.*0152*, *s*.*e*. = *0*.*0053*, *p* = *0*.*005*) and they did not survive the FDR correction threshold.

The Tukey post-hoc test revealed the significant enlargements in antidepressant-treated CHR-P and FEP subjects compared to non-treated CHR-P and FEP subjects in the surface of the *left fusiform gyrus (p* = *0*.*01)* and *the right inferior parietal lobule (p* = *0*.*048)*.

A group-wise Tukey post-hoc test registered the significant (*p* = *0*.*01*) increase in the surface of the medicated FEP group compared to the non-medicated FEP group in a right inferior parietal lobule and the non significant (*p* > *0*.*05*) trend to increase in the left fusiform gyrus, the left inferior temporal gyrus, the left inferior parietal lobule.

We found a significant interaction effect of antidepressant dosage and PSRS (Table 3) on the surface of the *precentral gyrus (p* = *0*.*0023)*, the *postcentral gyrus (p* = *0*.*0028)*, the *paracentral lobule (p* = *0*.*0014)* and the *lateral orbitofrontal cortex (p* = *0*.*0095)*. All interactions except in the *lateral orbitofrontal cortex* survived the FDR correction *in* 2 groups LME.

**Figure 1:**
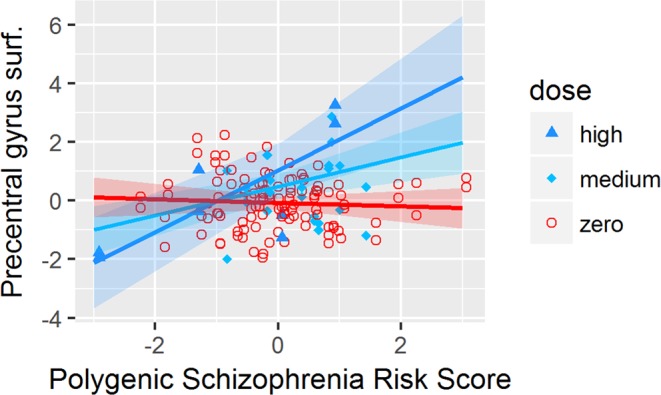
LME for significant interaction between antidepressants dosage and genomic risk score for the surface of the precentral gyrus.

**Figure 2:**
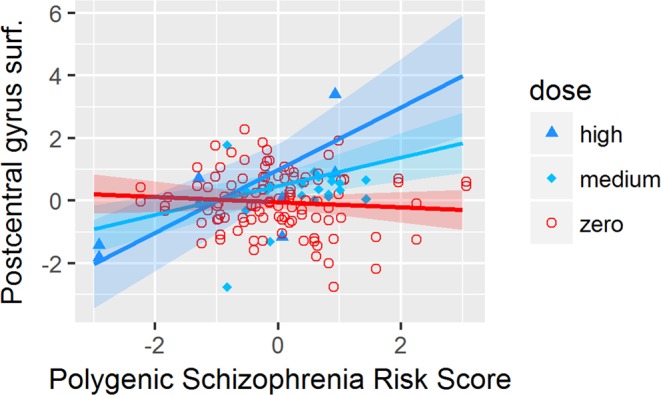
LME for significant interaction between antidepressants dosage and genomic risk score for the surface of the postcentral gyrus.

**Figure 3:**
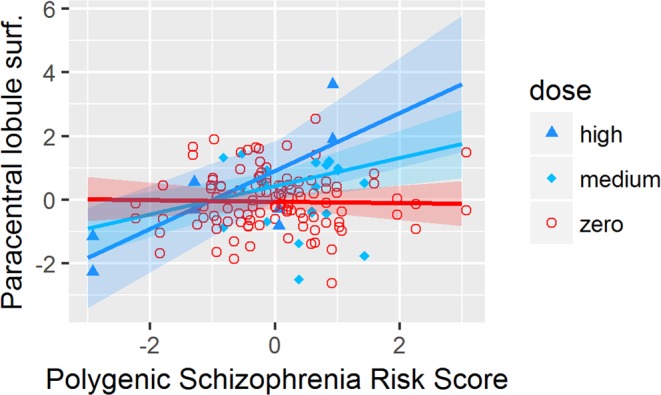
LME for significant interaction between antidepressants dosage and genomic risk score for the surface of the paracentral lobule.

**Figure 4:**
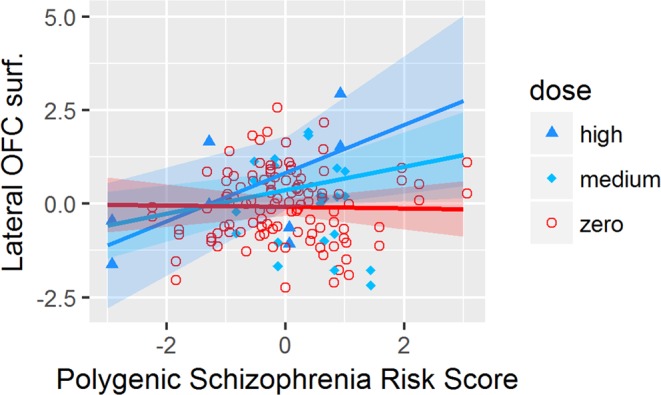
LME for significant interaction between antidepressants dosage and genomic risk score for the surface of the lateral orbitofrontal cortex.

Statistical comparison of the slopes of the LME lines (Figs 1–4) by a Tukey post-hoc test showed that there was a significant increase in the surface of the *bilateral precentral gyrus* (left and right *p* = *0*.*05;* Fig. 1), *right postcentral gyrus* (*p* = *0*.*004;* Fig. 2) and *left paracentral lobule* (*p* = *0*.*04;* Fig. 3) and a non-significant increase trend in the lateral orbitofrontal cortex *(p* > *0*.*05;* Fig. 4*)* in antidepressant-treated CHR-P and FEP subjects with increasing PSRS and current dose of antidepressants, but a slight decrease in non-treated CHR-P and FEP subjects.

### Cortical thickness

We found no significant main effects of antidepressant dosage on the cortical thickness of the analysed regions nor an interaction effect between antidepressant dosage and PSRS.

### LR tests

The LR tests *(*Tables S4 and S5*)* showed that the difference between the model pairs were significant (*p* < *0*.*01*) for all models reported in Tables 2 and 3, on the supposition that the antidepressant medication effect in the full sample and the interactions between PSRS and medication in the subsample were significant to explain variation in the data. The same test with 2 groups (CHR-P and FEP) excluding healthy controls confirmed our results for all models.

### Correlation analysis

The correlation analysis between the surface and volume of brain regions and the BPRS, SANS and GAF in the full sample and in the PSRS subsample (Tables S2 and S3) showed the negative correlations (*p* < *0*.*05 uncorrected*) in combined CHR-P and FEP subjects for *left fusiform gyrus* surface and BPRS-activation symptom subscale (*r* = −*0*.*207*), *left inferior temporal gyrus* surface and BPRS (*r* = −*0*.*251*), BPRS-activation (*r* = −*0*.*22*) and BPRS-positive (*r* = −*0*.*226*), *left middle temporal gyrus* surface with BPRS (*r* = −*0*.*232*) and BPRS-positive (*r* = −*0*.*235*), *left lateral OFC* surface with BPRS (*r* = −*0*.*309*), and with SANS (*r* = −*0*.*283*), *left paracentral lobule* surface and SANS (*r* = −*0*.*274*); *right inferior temporal gyrus* surface with BPRS-affective (*r* = −*0*.*176*), *right MTG* surface with BPRS-activation (*r* = −*0*.*199*), *right lateral OFC* surface with BPRS *(r* = −*0*.*265*), BPRS-positive (*r* = −*0*.*335*) and with GAF (*r* = *0*.*396*), *right precentral gyrus* surface with BPRS-affective (*r* = −*0*.*327*). The *left pallidum* volume was positively correlated with BPRS-negative subscale (*r* = *0*.*238*) and with SANS (*r* = *0*.*223*).

## Discussion

In an analysis of automatically segmented brain regions in CHR-P, FEP and healthy controls, we found a significant (FDR p < 0.05) main effect of current antidepressant dose on the volume of the *pallidum* and the *putamen* and surface of the *inferior temporal gyrus*. Furthermore, we found (FDR p < 0.05) that the surface of the *postcentral gyrus*, *the paracentral lobule* and *the precentral gyrus* can be linked to an interaction between antidepressants dosage and genomic risk score.

The volumes of the *bilateral putamen* and the *left pallidum* were enlarged in antidepressant-treated CHR-P and FEP compared to the untreated cohort and healthy controls. Diagnosis-related increases in the volume of *pallidum* and the *putamen* have been previously reported for schizophrenia and FEP patients^57,64,65^. *Putamen* and *pallidum* volume have been identified as predictors of positive symptoms and the duration of illness^52,64^ and it was shown that *putamen* lesions may lead to psychosis^66^. Moreover, the *basal ganglia* system has been associated with the dopaminergic hypothesis of schizophrenia^2^.

We found a significant main effect of antidepressant dose on the surface of the *inferior temporal gyrus*. In particular, the antidepressant-treated CHR-P and FEP subjects exhibited enlarged surface of the *left inferior temporal gyrus* compared to the non-treated CHR-P and FEP subjects. In patients with psychosis a decreased volume^67–71^ and surface area^72^ in the *inferior temporal gyru*s was demonstrated. Therefore, our finding suggests that antidepressant treatment might increase surface area of the inferior temporal gyrus in early stages of psychosis.

In the subsample with calculated PSRS, we found an interaction effect between antidepressant dose and PSRS on the surface of the *precentral* and the *postcentral* gyri, and the *paracentral lobule*. With the rise in both antidepressant dosage and PSRS, the *bilateral precentral gyrus*, the *right postcentral gyrus* and the *left paracentral lobule* increased in surface - compared to non-treated CHR-P and FEP subjects, where a decreasing trend appeared.

Morphological and functional changes in these regions have been previously associated with psychosis: reduced surface area in the right postcentral gyrus have been reported in 22q11DS subjects^73^ and patients with schizophrenia revealed significant reduction in surface area in frontal and parietal regions;^74^ the displacement^75^, abnormal activation^76^ and the reduction^77,78^ of the *postcentral gyrus* are characteristic for psychosis.

The regions where the increase in PSRS is linked to the surface increase due to antidepressant treatment are connected to important clinical symptoms. The precentral gyrus has been associated with verbal hallucinations^79^, neurocognitive deficits and attentional deficits^80^. Connectivity changes in the *postcentral gyrus* with the *dorsolateral prefrontal cortex* are linked to the improvement in affective psychotic symptoms^81^.

According to our findings, one could suggest that antidepressant medication might have a stronger influence on surfaces than thicknesses. Their relation is contradictory, as there is evidence that they’re negatively correlated^82^, but also that they’re genetically unrelated and develop independently^83^. This is in agreement with our finding that only surface but not thickness shows an interaction between antidepressants and PSRS. So the effect of antidepressants might be genetically determined and only evident for surfaces. Future research is warranted whether the effect of antidepressants in FEP/CHR-P is specific for surfaces and whether it is determined by genetic predisposition.

The overall improvements in CHR-P and schizophrenia after antidepressant treatment reported in the literature are quite inconsistent^84,85^. According to the study of remission from major depression^86^, remitters show increase over time and nonremitters show decrease in volume and thickness of cortical and subcortical structures.

The relationship between antidepressant dosage and morphology identified in our study should be further examined to determine whether antidepressants can efficiently alleviate symptoms and which dose is required; some efforts are already ongoing^87^. Thus, determination of the dosage, start of prescription and combination with other drugs might be improved in subjects with emerging psychosis.

In further studies we consider using the duration of antidepressant admission because we cannot exclude a possibility that those receiving antidepressants may represent a different subpopulation.

The evidence of antidepressant effects on the morphophysiology of regions that have a possible link to clinical symptoms may provide a scientific rationale to support the notion that earlier antidepressant prescription for CHR-P patients could improve their clinical outcomes^34^. As the regional enlargements are negatively correlated to symptoms, the interactions between PSRS and antidepressant dose found in our pilot study of the PSRS subsample might also be of clinical interest as we could speculatively propose that subjects with higher PSRS may benefit more from antidepressant treatment in order to reduce early symptoms and to limit the morphophysiological side effects. Thus, the translational significance of the findings is that these associations may influence the clinical choice of optimal medication in CHR-P and FEP subjects.

Our study had some limitations. Differences in cannabis consumption among groups may also have been influenced our findings, although the effect of cannabis on morphological brain measures in healthy volunteers and psychotic patients is inconclusive^88–91^. It was not possible to calculate the cumulative lifetime treatment dose of antidepressants due to gaps in medical records. Nevertheless, further studies should include the lifetime dose, as mean dose provides incomplete information in cross-sectional paradigms. The mean antidepressant dose used in the clinic is relatively low and due to the presence of agents with a mixed range of pharmacological action and the limited sample size, we were unable to differentiate groups based on antidepressant types. Our sample size did not allow a meaningful statistical subanalysis to differentiate between the different clinical high-risk subgroups APS, BLIPS and GRD^9^. Given that their risk of developing psychosis is different^13^, future studies are advised to stratify their findings across these subgroups. The number of tests was large due to the multitude of regions possibly implicated in response to antidepressant treatment. The current inferences about the PSRS effect are speculative because of the modest sample, and the future studies should investigate different morphophysiological effects in low and high PSRS cohort, as more genetic data should be collected. The effect of disease stage should also be included in the larger sample. The underlying biological mechanism of these modifications needs to be further investigated.

In sum, to our knowledge, we have demonstrated for the first time the association of the converted antidepressant dosage on morphological brain changes and the interaction between antidepressants dosage and genomic risk score in CHR-P and FEP subjects. As most of the reported regions were shown as linked to the clinical symptoms of psychosis, our findings may contribute to explanations of the suggested beneficial effects of antidepressant treatment in this population.

## Methods

### Participants

We use the cross-sectional data of 142 patients (72 FEP, 70 CHR-P) and 55 controls recruited in the FePsy (early detection of psychosis) study (see full *Participants*, *Screening* and *Genotyping* description in *Supplementary materials*). The PSRS data was obtained for a subsample of 61 patients: 32 FEP and 29 CHR-P, as well as for 14 controls. The data overlaps (n = 72) with our previous studies^57^.

All participants provided written informed consent and received compensation for participating. The studies had permission from the ethics committee beider Basel (EKBB). All methods were performed in accordance with the relevant guidelines and regulations.

### Screening

The participants were assessed using the Basel Screening Instrument for Psychosis (BSIP)^92^. Subjects selected for the study by screening subsequently underwent an entry examination, which included the Brief Psychiatric Rating Scale (BPRS), Scale for the Assessment of Negative Symptoms (SANS), and a neuropsychological test battery. Inclusion criteria in the CHR-P group required (a) attenuated symptoms or (b) brief limited intermittent psychotic symptoms and genetic risk or schizotypal features, coupled with functional deterioration^93^. The transition to FEP required the occurrence of at least one positive psychotic symptom several times a week for a continuous period of time.

### Exclusion criteria

Age below 18 years, insufficient knowledge of German, IQ < 70, previous episode of psychosis treated with major tranquillisers for >3 weeks, a psychosis due to organic reasons or substance abuse, or psychotic symptomatology within a clearly diagnosed affective psychosis or borderline personality disorder.

### Medication

The current mean converted antidepressants dose was 28.14 mg *Fluoxetine equivalent*^94^ in the full sample and 29.53 mg for the PSRS subsample. The assignment of antidepressant treatment in our sample was non-random i.e. according to clinical needs and consisted of escitalopram (n = 10), fluoxetine (n = 4), citalopram (n = 2), paroxetine (n = 2), sertraline (n = 2); venlafaxine (n = 4); mirtazapine (n = 5); trazodone (n = 2) and bupropion (n = 1). The PSRS subsample included escitalopram (n = 3), fluoxetine (n = 4), sertraline (n = 1); venlafaxine (n = 4); mirtazapine (n = 2) and trazodone (n = 2) treatment. N = 38 subjects from the full sample and n = 12 subjects from PSRS subsample were medicated with second-generation antipsychotics (SGA). Current antipsychotic dose was converted into chlorpromazine (CPZ) equivalents^95^. The mean CPZ equivalents (s.d.) were 214.5 (271.1). N = 9 subjects were taking both SGA and antidepressants. No healthy control was medicated with antidepressants or SGA. N = 36 subjects from the full sample and n = 18 subjects from PSRS subsample consumed cannabis.

### Genotyping and PSRS calculation

DNA in the PSRS subsample was extracted from whole-blood samples. PSRS was calculated by taking linkage disequilibrium-pruned loci^58,96^. A total of 87 SNPs that could be mapped to one of the top SNPs of the 108 loci associated with schizophrenia and that survived quality control were used to calculate the PSRS. The number of risk alleles per person was weighted for each SNP by the logarithm of its odds ratio as reported in the PGC SZ data set and summed across SNPs^97^. The PSRS was then corrected for the first 20 genotypic principal components (PCs) and the number of SNPs used to calculate the PSRS by using the z-transformed residuals of a linear regression.

### Acquisition and analysis of MRI data

We obtained structural MRI scans within an average of 25 days after entry into our early detection service, using a 3 T MR imaging scanner (*Siemens Magnetom Verio*, *Siemens Healthcare*, *Erlangen*, *Germany*) with a 12-channel phased-array radio frequency head coil. For structural images, a 3D T_1_-weighted magnetisation-prepared rapid gradient echo sequence was used with the following parameters: inversion time: 1000 ms, flip angle = 8 degrees, repetition time = 2 s, echo time = 3.37 ms, field of view = 25.6 cm, acquisition matrix = 256 × 256 × 176, resulting in 176 contiguous sagittal slices with 1 × 1 × 1 mm^3^ isotropic spatial resolution. All scans were screened for gross radiological abnormalities by an experienced neuroradiologist. N = 16 subjects were excluded due to erroneous MR scans (2 FEP, 11 CHR-P and 3 HC).

MR images were processed through Freesurfer 6.0 automated segmentation pipeline (https://surfer.nmr.mgh.harvard.edu/fswiki/recon-all/). A total of 41 parcellated brain regions were obtained using the *recon-all* fully-automated directive workflow with the default Deskian-Killiany atlas. The workflow included motion correction, brain extraction, Talairach transformation, segmentation of cortical and subcortical structures, intensity normalization, gray matter-white matter boundary tessellation, and topology correction. Results were visually inspected and statistically evaluated for outliers following standardized ENIGMA protocols for cortical and subcortical structures (http://enigma.ini.usc.edu/protocols/imaging-protocols/) and outlier removal was performed with the code provided by the ENIGMA Consortium (http://enigma.ini.usc.edu/protocols/imaging-protocols/) and continued if the regional value was not in a range of ±3.5 standard deviations. Subsequently, cortical thickness, surface area and subcortical volume of all 41 brain areas were normalised with respect to intracranial volume and centred.

After the quality check of the main study population (Table 1), the full sample, with and without PSRS data, consisted of combined 22 CHR-P and 11 FEP subjects treated with antidepressants, 45 CHR-P and 57 FEP individuals without antidepressant treatment and 55 healthy controls.

### Statistical analysis

The *R software v*.*3*.*4*.*0* with packages ‘*lmertest*’*v*.*2*.*0*.*36*, ‘*effects*’*v*.*4*.*0*, ‘*emmeans*’*v*.*0*.*9*.*1*, ‘*car‘ v*.*2*.*1*.*6* was used for statistical, group-related descriptive analysis. Adequate statistical tests (ANOVA, logistic regression, chi-squared test or t-test) were applied to examine group effects on the following variables of interest: age, sex, handedness, years of education, IQ, BPRS, PSRS, cannabis use, antipsychotics and antidepressants (Tables 1 *and* S1). Additionally the Pearson correlation coefficient was calculated for BPRS^98^, SANS and GAF score correlated with the anatomical data of each separate region. Current mean antidepressant dose was converted into fluoxetine equivalents^94^.

To investigate the medication effect on brain morphology (cortical thickness, surface area and subcortical volume), we constructed several linear mixed effect models (LME) that contained current converted antidepressant medication dose, diagnosis, sex, age, hemisphere, and interaction between current daily medication dose and hemisphere as fixed effects and intercept for every subject as random effect for each brain region. To test the hypothesis that antidepressant medication effects on cortical and subcortical structures varies in subjects with different genetic predisposition for psychosis, we constructed LME that included current converted medication dose of antidepressants, corrected PSRS score, interaction between daily medication dose and PSRS, diagnosis, sex, age, hemisphere and interaction between medication, PSRS and hemisphere as fixed effects and intercept for subject as random effect. LMEs were summarized by an ANOVA type 2 for medication effects alone (Table 2*)* and by an ANOVA type 3 for the interaction effects between medication and PSRS score in the PSRS subsample (Table 3).

Analyses for each region were followed by Tukey’s HSD post-hoc test uncontrolled for 41 brain regions studied (Tables S6–S8, p < 0.05 was considered significant*)*. Likelihood ratio (LR) tests were performed for all of the constructed LME in the corresponding brain regions (Tables S4 and S5). Reduced models did not contain [*medication*] and [medication*hemisphere] effects in the full sample and did not contain the interactions [PSRS*medication] and [PSRS*medication*hemisphere] in the PSRS subsample.

## Supplementary information

Supplementary Info
