# Supplementary material for: Association of antidepressants with brain morphology in early stages of psychosis: an imaging genomics approach
Source: Sci Rep. 2019 Jun 11;9:8516. doi: 10.1038/s41598-019-44903-y (PMC6560086; doi:10.1038/s41598-019-44903-y)
Supplement: Supplementary file 1 — Supplementary Info [file 41598_2019_44903_MOESM1_ESM.docx]

**Title:** Association of antidepressants with brain morphology in early stages of psychosis: an imaging genomics approach

**Author list:** Oleg Bykowsky, Fabienne Harrisberger, André Schmidt, Renata Smieskova, Daniel J. Hauke, Laura Egloff, Anita Riecher-Rössler, Paolo Fusar-Poli, Christian G. Huber, Undine E. Lang, Christina Andreou, Stefan Borgwardt*

*Corresponding author

**Participants**

The participants were recruited in the *FePsy* (early detection of psychosis) study^1,2^, a multi-domain study which was set up specifically to identify, assess and treat individuals in the early stages of psychosis. Subjects with CHR-P or FEP were recruited from a service area covering about 200 000 inhabitants in and around Basel, Switzerland, through an early detection service of the FePsy Clinic University Psychiatric Hospital Basel.

**Screening**

The CHR-P, FEP and healthy controls were assessed using the Basel Screening Instrument for Psychosis (BSIP), specifically developed for this purpose^3^. It was constructed as a screening checklist to identify those at risk for psychosis and is followed by a more extensive early detection interview in a next assessment step. It is not a screening instrument to be used in the general population, but in help-seeking populations and has to be performed by experienced psychiatrists. This instrument is based on the prodromal symptoms and other prodromes as derived from literature such as social decline, drug abuse, previous psychiatric disorders, or genetic risk; four psychosis items of the expanded version of the Brief Psychiatric Rating Scale (BPRS) ^4,5^ for assessing (pre-) psychotic phenomena are incorporated. The BSIP allows the rating of individuals regarding the inclusion/exclusion criteria corresponding to the PACE criteria ^6,7^ and has been shown to have a good inter-rater reliability (κ=.67) and a high predictive validity ^3^.

Interview measures for a CHR such as the Comprehensive Assessment of At-Risk Mental States (CAARMS), the Structured Interview for Prodromal Syndromes (SIPS) and the companion Scale of Prodromal Symptoms (SOPS), and the Basel Screening Instrument for Psychosis (BSIP). The CAARMS was developed by Yung and Colleagues ^14^ at the PACE clinic in Melbourne and has been widely used in Australia, Asia and Europe. The BSIP was developed in the Early Detection of Psychosis Clinic (FEPSY) in Basel by Riecher et al.^3^ McGlashan and colleagues^15^

developed the SIPS/SOPS, which have become the instruments most used in North American studies. All 3 instruments are semistructured interviews and assess similar clinical features.^16^

All subjects selected for the study by screening subsequently underwent an extensive entry examination, which included the BPRS^4^, Scale for the Assessment of Negative Symptoms (SANS), and a neuropsychological test battery.

**Genotyping**

DNA was extracted from whole-blood samples using the QIAamp DNA Blood Maxi kit according to the standard procedures (Qiagen, Chatsworth, CA, USA). DNA samples were further processed on the Affymetrix Genome-Wide Human SNP Array 6.0. in one centralised microarray facility as described in the Genome-Wide Human SNP Nsp/Sty 6.0. User Guide (Affymetrix, Santa Clara, CA, USA). Generation of SNP calls and array quality control (QC) were performed using the Affymetrix Genotyping Console Software 3.0 (Affymetrix). According to the manufacturer’s recommendation, contrast QC was chosen as QC metric, using the default value of 0.4. All samples passing QC criteria were subsequently genotyped using the Birdseed (v2) algorithm, leading to a total of 921 523 genotyped SNPs per sample. Appropriate SNP QC filtering was applied in the PLINK 1.9 software^8,9^ where the gender check in PLINK led to the exclusion of three individuals.

Population stratification was assessed using principal component (PC) analysis implemented in the EIGENSTRAT software^10^ to detect genotypic outliers (with default parameters: >6 s.d.'s on any of the top 10 PCs in five iterations) and to correct for the potential population substructure by analysing all array-based pruned, autosomal SNPs. Eight individuals were identified as outliers and therefore were excluded from further analyses.

Before autosome-wide genotype imputation, haplotype estimation was performed using SHAPEITv2 software^11^ allowing a per individual and a per SNP missing rate for observed markers of max. 5%. After pre-phasing, genotype imputation was performed using IMPUTE v2.3.0 software, which imputes missing genotypes using a multipopulation reference panel^12,13^. The integrated variant callset of 1092 individuals from the 1000 Genomes Project (release v3 in NCBI build 37/hg19 coordinates, March 2012) served as panel data (http://mathgen.stats.ox.ac.uk/impute/ALL_1000G_phase1integrated_v3_impute_macGT1.tgz). Only genotype calls exceeding a probability score of 90% were converted into genotype calls for statistical analysis using the PLINK 1.9 software.^8^

**Supplementary references**

1. **Riecher‐Rössler A et al**. The Basel early-detection-of-psychosis (FEPSY)-study-design and preliminary results. Acta Psychiatrica Scandinavica. Feb; 115(2):114-25, https://doi.org/10.1111/j.1600-0447.2006.00854.x (2007)
2. **Riecher-Rössler A et al.** Efficacy of Using Cognitive Status in Predicting Psychosis: A 7-Year Follow-Up. Biological Psychiatry, 66(11), 1023-1030, https://doi.org/10.1016/j.biopsych.2009.07.020 (2009)
3. **Riecher-Rössler A et al.** The Basel Screening Instrument for Psychosis (BSIP): development, structure, reliability and validity. Fortschritte der Neurologie-Psychiatrie; 76: 207–216, https://doi.org/10.1055/s-2008-1038155 (2008)
4. **Lukoff D, Nuechterlein KH, Ventura J**: Manual for the expanded brief psychiatric rating scale. Schizophrenia Bulletin 12:594–602(1986)
5. **Ventura J et al.**: Training and quality assurance with the brief psychiatric rating scale: “The Drift Busters”; Appendix 1 the Brief Psychiatric Rating Scale (expanded version). International Journal of Methods in Psychiatric Research 3:221–224 (1993)
6. **Yung AR et al**. Prediction of psychosis: a step towards indicated prevention of schizophrenia. The British Journal of Psychiatry. Jun;172(S33): 14-20, https://doi.org/10.1192/s0007125000297602 **(**1998**)**
7. **Yung AR et al.** PACE: A specialised service for young people at risk of psychotic disorders. Medical Journal of Australia 187: 43–46 (2007)
8. **Purcell S et al.** PLINK: a tool set for whole-genome association and population-based linkage analyses. Am J Hum Genet. Sep; 81(3):559-75, https://doi.org/10.1086/519795 (2007)
9. **Chang CC et al**. Second-generation PLINK: rising to the challenge of larger and richer datasets. GigaScience; 4: 7, https://doi.org/10.1186/s13742-015-0047-8 (2015)
10. **Price AL et al.** Principal components analysis corrects for stratification in genome-wide association studies. Nat Genet. Aug; 38(8):904-9, https://doi.org/10.1038/ng1847 (2006)
11. **Delaneau O, Zagury JF, Marchini J**. Improved whole-chromosome phasing for disease and population genetic studies. Nat Methods. Jan; 10(1):5-6, https://doi.org/10.1038/nmeth.2307 (2013)
12. **Howie B, Marchini J, Stephens MG**. Genotype imputation with thousands of genomes. Nov; 1(6):457-70, https://doi.org/10.1534/g3.111.001198 (2011)
13. **Howie BN, Donnelly P, Marchini J**. A flexible and accurate genotype imputation method for the next generation of genome-wide association studies. PLoS Genet. Jun; 5(6), https://doi.org/10.1371/journal.pgen.1000529 (2009)
14. **Yung, A.R., et al.** Mapping the onset of psychosis: the comprehensive assessment of at‐risk mental states. Australian and New Zealand Journal of Psychiatry 39.11‐12, 964-971, https://doi.org/10.1080/j.1440-1614.2005.01714.x (2005)
15. **Miller, T.J., et al.** Prodromal assessment with the structured interview for prodromal syndromes and the scale of prodromal symptoms: predictive validity, interrater reliability, and training to reliability. Schizophrenia bulletin 29.4: 703-715, https://doi.org/10.1093/oxfordjournals.schbul.a007040 (2003)
16. **Fusar-Poli, P., et al.** Predicting psychosis: meta-analysis of transition outcomes in individuals at high clinical risk. Arch. Gen. Psych. 69.3, https://doi.org/10.1001/archgenpsychiatry.2011.1472, 220-229 (2012)
